# *In situ* community transcriptomics illuminates CO_2_-fixation potentials and supporting roles of phagotrophy and proton pump in plankton in a subtropical marginal sea

**DOI:** 10.1128/spectrum.02177-23

**Published:** 2024-02-06

**Authors:** Hongfei Li, Jianwei Chen, Liying Yu, Guangyi Fan, Tangcheng Li, Ling Li, Huatao Yuan, Jingtian Wang, Cong Wang, Denghui Li, Senjie Lin

**Affiliations:** 1 State Key Laboratory of Marine Environmental Science, Xiamen University, Xiamen, Fujian, China; 2 National Engineering Research Center for Marine Aquaculture, Zhejiang Ocean University, Zhoushan, Zhejiang, China; 3 Department of Marine Sciences, University of Connecticut, Groton, Connecticut, USA; 4 Qingdao Key Laboratory of Marine Genomics, BGI Research, Qingdao, Shandong, China; 5 State Key Laboratory of Agricultural Genomics, BGI Research, Shenzhen, Guangdong, China; 6 Qingdao Innovation Center of Seaweed Biotechnology, Qingdao, Shandong, China; University of Mississippi, University, Mississippi, USA

**Keywords:** whole-assemblage metatranscriptomics (WAME), phytoplankton, microbial, carbon fixation, proton-pump rhodopsin, dinoflagellates

## Abstract

Lineage-wise physiological activities of plankton communities in the ocean are important but challenging to characterize. Here, we conducted whole-assemblage metatranscriptomic profiling at continental shelf and slope sites in the South China Sea to investigate carbon fixation potential in different lineages. *RuBisCO* expression, the proxy of Calvin carbon fixation (CCF) potential, was mainly contributed by Bacillariophyta, Chlorophyta, Cyanobacteria, and Haptophyta, which was differentially affected by environmental factors among lineages. CCF potential exhibited positive or negative correlations with phagotrophy gene expression, suggesting phagotrophy possibly enhances or complements CCF. Our data also reveal significant non-Calvin carbon fixation (NCF) potential, as indicated by the active expression of genes in all five currently recognized NCF pathways, mainly contributed by Flavobacteriales, Alteromonadales, and Oceanospirillales. Furthermore, in Flavobacteriales, Alteromonadales, Pelagibacterales, and Rhodobacterales, NCF potential was positively correlated with proton-pump rhodopsin (PPR) expression, suggesting that NCF might be energetically supported by PPR. The novel insights into the lineage-differential potential of carbon fixation, widespread mixotrophy, and PPR as an energy source for NCF lay a methodological and informational foundation for further research to understand carbon fixation and the trophic landscape in the ocean.

**IMPORTANCE:**

Marine plankton plays an important role in global carbon cycling and climate regulation. Phytoplankton and cyanobacteria fix CO_2_ to produce organic compounds using solar energy and mainly by the Calvin cycle, whereas autotrophic bacteria and archaea may fix CO_2_ by non-Calvin cycle carbon fixation pathways. How active individual lineages are in carbon fixation and mixotrophy, and what energy source bacteria may employ in non-Calvin carbon fixation, in a natural plankton assemblage are poorly understood and underexplored. Using metatranscriptomics, we studied carbon fixation in marine plankton with lineage resolution in tropical marginal shelf and slope areas. Based on the sequencing results, we characterized the carbon fixation potential of different lineages and assessed Calvin- and non-Calvin- carbon fixation activities and energy sources. Data revealed a high number of unigenes (4.4 million), lineage-dependent differential potentials of Calvin carbon fixation and responses to environmental conditions, major contributors of non-Calvin carbon fixation, and their potential energy source.

## INTRODUCTION

The functional contribution of individual lineages to a marine plankton community is challenging to characterize and has been underexplored. Traditional taxonomic composition analysis provides community structure but does not offer information on the physiological performance of each taxon. Physiological measurements, on the other hand, reveal the functional activities of the community but cannot resolve the contribution of individual taxa. Metatranscriptomic analysis can fill this gap because expressed genes in the resulting data set can be traced back to the source lineage of organisms, whereas the expression levels of the genes (proxies of the potential activities of the encoded functions) can be quantified and compared between lineages. Thanks to the rapid growth of genomic and transcriptomic databases and the increasing accessibility of metatranscriptome sequencing and bioinformatic analyses, metabolic profiles in dinoflagellates (1–3), diatoms (4), raphidophytes (5), and other lineages (4) in the natural marine environment have increasingly been documented. The metatranscriptomic approach has enabled the interrogation of the potential to acquire energy and nutrients, defend against grazing and microbial attacks, and perform sexual reproduction of individual phytoplankton species in the community, with which to understand the controlling drivers of regime shifts in the community (6–12). Most of these studies have focused on one or a few species in the communities, and cross-kingdom or domain comparative insights on multi-metabolic pathways have only begun to emerge and are still relatively limited. Carradec and colleagues (13) reported the first global ocean atlas of eukaryotic genes based on the *Tara Oceans* expedition. Recently, a study in the tropical Pacific revealed that dinoflagellates occupy an important niche by employing numerous metabolic strategies and play a dual role in carbon transformation (14). A metatranscriptomic study on a dinoflagellate bloom characterized the differential metabolic potential of phytoplankton, zooplankton, bacteria, and viruses and harnessed insights into how the bloom dynamics of *Karenia longicanalis* was modulated by bottom-up (nutrients and energy) and top-down (grazing and microbial attacks) processes (12). More whole-assemblage (covering eukaryotes and prokaryotes) studies are needed, especially focusing on lineage-specific contributions to carbon fixation.

Calvin carbon fixation (CCF) is usually dominated by cyanobacteria in the oligotrophic ocean and by diatoms and other eukaryotes in the coastal waters (15, 16). Non-Calvin carbon fixation (NCF) by bacteria has been anecdotally documented or discussed but has not become a focused topic of research (17–19). NCF involves five metabolic pathways and has been suggested to potentially contribute as high as >30% of total oceanic carbon fixation as it can be performed both at night (dark C fixation) and under light (20). Although NCF was increasingly reported for marine bacterial isolates in recent years (17, 18, 21), lineage-specific activities of NCF and their contribution to total carbon fixation are still poorly understood. Besides, extensive laboratory studies indicate that many planktonic eukaryotes are capable of mixotrophy, using different degrees of phagotrophy combined with complementary degrees of photoautotrophy (22, 23). A recent study in the tropical Pacific indicates that phagotrophy of dinoflagellates is important in euphotic and the mesopelagic zones (14). How active individual lineages are in carbon fixation and mixotrophy, and what energy source bacteria may employ in NCF in natural plankton assemblages are poorly understood and underexplored.

In this study, we conducted a whole-assemblage metatranscriptomic (WAME) study for a continental shelf station and a continental slope station in the South China Sea (SCS) to gain a comprehensive understanding of lineage-specific functional contributions. SCS is a subtropical marginal sea that connects the Tibetan Plateau and the Western Pacific Warm Pool. There are both terrestrial input from the plateau and exchange from the warm ocean currents (24, 25). The coastal waters (within the continental shelf) are influenced by several major rivers, which bring in high nutrient loads (26, 27). The basin of the SCS (continental slope and beyond), however, is generally oligotrophic, even though it receives nutrient inputs from the seasonally fluctuated Kuroshio current, upwelling, and monsoons (28, 29). Its carbon biogeochemistry has been extensively investigated, and parts of the ocean are found to be carbon sinks, whereas other parts are carbon sources, at least seasonally (30). By cataloging the functional (i.e., protein-coding) gene repertoire, characterizing metabolic pathways in different lineages, comparing the potentials of CCF, characterizing phagotrophic activities, and assessing NCF activity and energy sources, we aim to shed light on the metabolic dynamics and carbon cycling in the diverse plankton assemblages of the SCS. Our data set reveals a vast number of unigenes (4.4 million) and provides insights into the lineage-specific activities of carbon fixation and the potential sources of energy for different lineages.

## RESULTS

### Retrieved genes and their functional and taxonomic distributions

**Fig 1:**
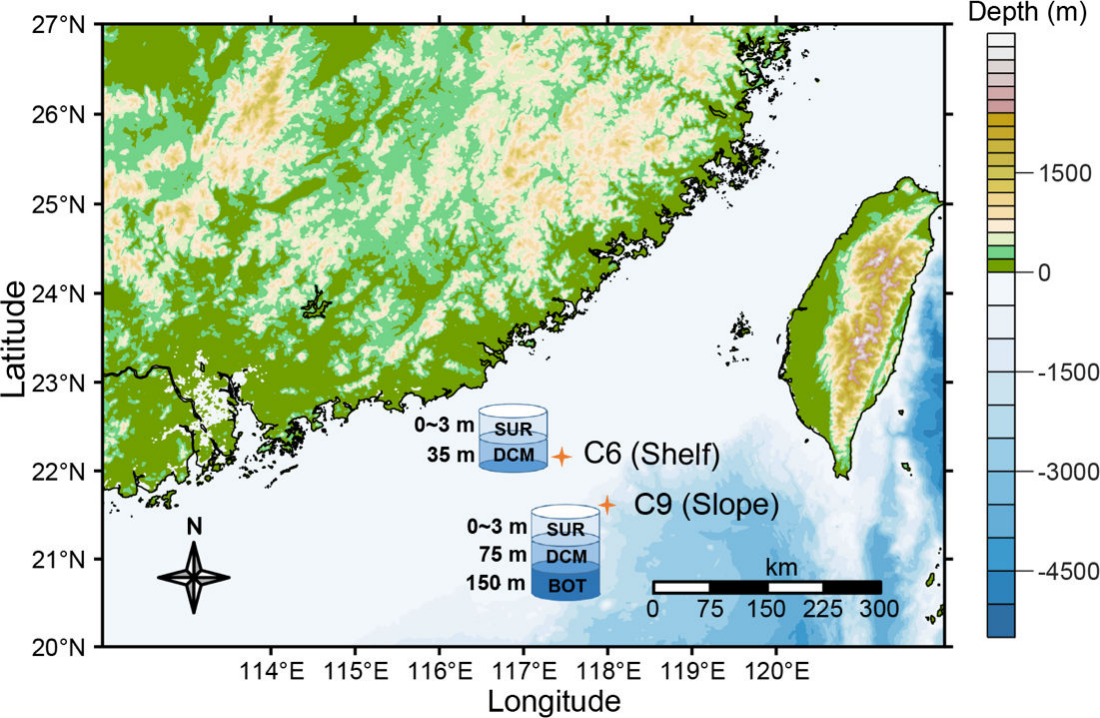
Study sites and sampling schemes. A continental shelf site (C6) and a continental slope site (C9) were sampled for the depths of surface (SUR) and deep chlorophyll maximum (DCM), and for C9, also the 150-m depth defined as the bottom of the euphotic zone (BOT). Station C6 (117.46°E, 22.13°N) is on the continental shelf, with a depth of 77 m, therefore, had no samples for 150 m. Station C9 (117.99°E, 21.69°N) is on the continental slope, with a depth of 1,369 m. For each sample, 40–50 L of water was collected and serially filtered onto 0.2–3 and 3–200 µm size fractions.

The 20 samples from which WAME was analyzed were collected from two to three different depths in picoplankton (0.2–3 µm) and nano-/micro-plankton (3–200 µm) organismal size fractions, at two stations, hence corresponding to 10 different conditions, with two biological replicates for each condition (Fig. 1). Physical and chemical parameters are shown in Table 1, showing a temperature range of 18.61°C–30.25°C, salinity of 33.62–34.78, N-nutrients 0.16–9.11 µM, and P-nutrient 0.01–0.72 µM. WAME sequencing and bioinformatics of these samples yielded a total of 881 Gb of raw reads, which were assembled into 4,499,414 unigenes, with *N*_50_ of 372 bp and a maximum length of 71,092 bp (Table 2). Rarefaction analysis indicated that our sequencing effort was close to saturation to recover all expressed genes (Fig. S1). Of these unigenes, 37.23% matched genes that have functional annotations in the databases (Table S1). These unigenes were contributed by 26,269 taxa of organisms (Table S2). It is important to note that the data and the metabolic profiles reported here only represent a snapshot of the potentially very dynamic and heterogeneous plankton communities. The actual numbers of unigenes and contributing lineages as well as their metabolic profiles remain to be portrayed with more spatially and temporally intensive sampling schemes in future research. Nevertheless, the insights gained from the limited data are still worth discussing.

**TABLE 1:** Physical and chemical parameters

	C6S_1	C6S_2	C6D_1	C6D_2	C9S_1	C9S_2	C9D_1	C9D_2	C9B_1	C9B_2
NO_2_^−^ + NO_3_^−^ (μm)	0.3985	0.272	1.2915	0.281	0.607	0.156	0.584	2.291	9.108	8.246
PO_4_^−^ (μm)	0.058	0.053	0.538	0.087	0.011	0.021	0.131	0.242	0.717	0.648
SiO_3_^2−^ (μm)	3.0145	2.065	2.7505	1.557	1.527	1.497	2.702	3.61	8.641	7.769
Temperature (°C)	29.26	28.5935	27.0803	27.0055	30.2508	29.8893	24.5048	26.2942	18.61	18.9146
Salinity	33.62	34.2943	34.1409	34.4909	34.0557	33.7776	34.6454	34.7504	34.7678	34.7839
Depth (m)	3	3	35	35	3	3	75	75	150	150
Photosynthetically active radiation (μmol m^−2^ s^−1^)	55.8697	55.8697	4.007	4.007	59.0434	59.0434	0.7454	0.7454	0.0221	0.0221

**TABLE 2:** Statistics of non-redundant gene sets

Statistics	
Total number (#)	4,449,414
Total length (bp)	1,669,935,941
Gap (*N*) (bp)	0
Average length (bp)	375.32
*N*_50_ length (bp)	372
*N*_90_ length (bp)	226
Maximum length (bp)	71,092
Minimum length (bp)	200
GC content	44.99%

### CCF in different plankton groups and environmental effects

**Fig 2:**
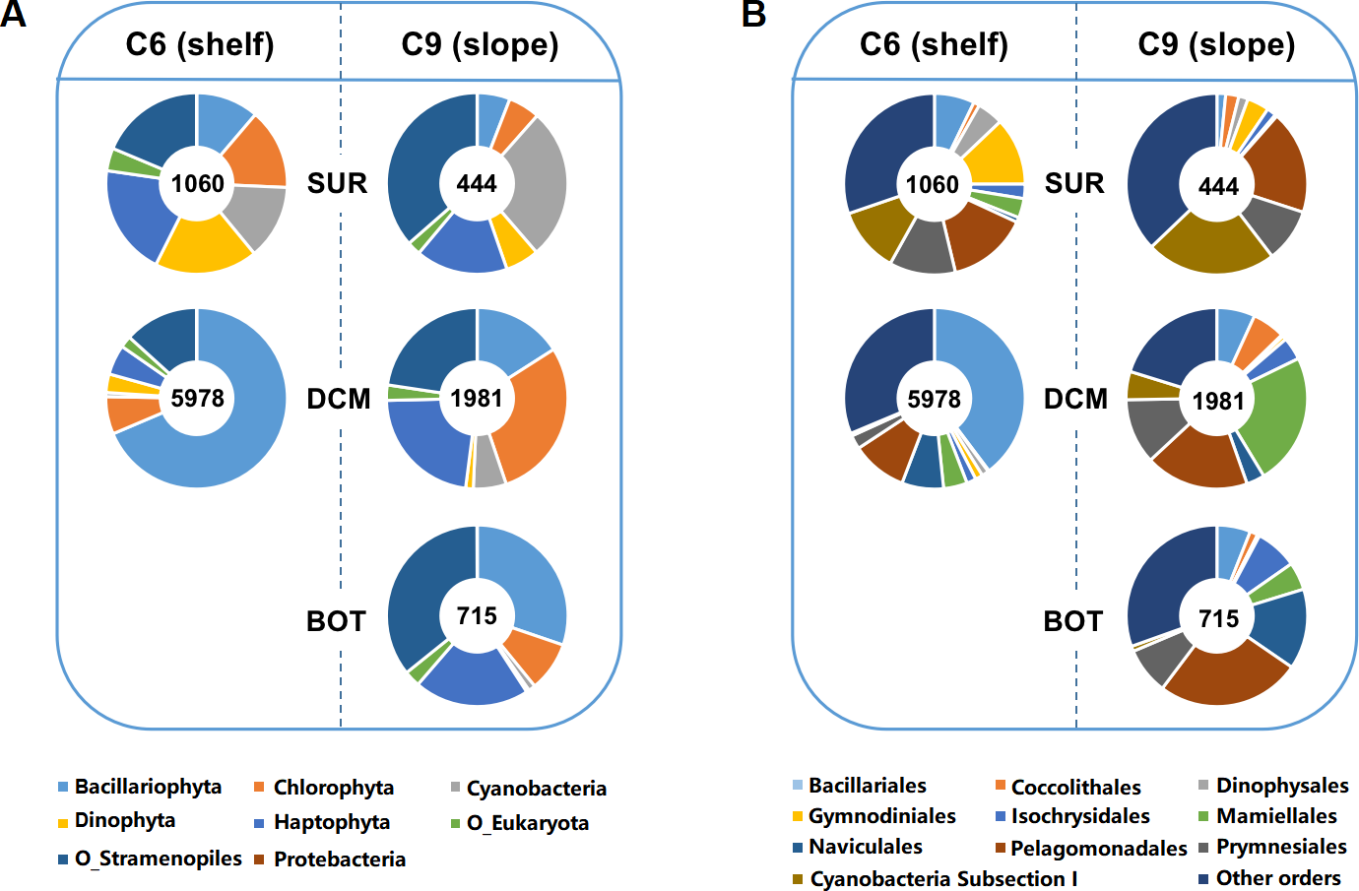
Relative contributions of major lineages to the CCF transcript pool in different water layers. (A) Comparison at supergroup level; number in the inner circle depicts the expression level (transcripts per kilobase of exon model per million mapped reads) of *RuBisCO* gene. (B) Comparison at order level.

*RuBisCO* gene expression is the rate-limiting enzyme of photosynthetic carbon fixation and thus was used as a proxy to compare the potential of CCF in plankton. Among the samples, the relative CCF potential in the deep chlorophyll maximum (DCM) layer was higher than the surface layer (SUR) and bottom of the photic zone (BOT) at both stations, with the DCM of C6 station (shelf) being the highest of all sample sources. The relative CCF potential in the continental shelf area was higher than that in the continental slope area with the same water layer, and that in the SUR layer of C9 the station (slope) was the lowest of all sample sources (Fig. 2A).

At the supergroup level, the main contributors of CCF transcript pool were Bacillariophyta, Haptophyta, Cyanobacteria, Chlorophyta, and non-diatom Stramenopiles (O_Stramenopiles) (Fig. 2A). In the SUR layer at C6 station, Proteobacteria and Other Eukaryota (O_Eukaryota) contributed a small proportion of CCF transcripts, while other supergroups have similar proportions, although Haptophyta contributed slightly more than other supergroups. In the DCM layer of the shelf, the dominant CCF transcript contributor was diatoms (Bacillariophyta), which accounted for 68.60% of the total CCF potential. In the SUR layer of the slope, the main CCF transcript contributors were O_Stramenopiles, Cyanobacteria, and Haptophyta, which accounted for 36.46%, 27.15%, and 16.43% of total CCF potential, respectively. In this study, cyanobacteria contribution was the greatest in the slope_SUR environment. Bacillariophyta also contributed substantially to the CCF of the BOT layer at slope, accounting for 30.23%, and other stramenopile phyla overwhelmingly dominated the total CCF potential. For CCF-contributing Proteobacteria, only one species named *Azoarcus kh32c* was represented in the WAME data set in the entire study, and its *RuBisCO* expression was detected only in the BOT layer of the slope, contributing 0.04% to the total CCF potential.

For Bacillariophyta, the relative contributions to CCF potential in the surface layer and DCM layer in the shelf region were 11.24% and 68.60%, respectively. In the slope region, the relative contributions in the surface layer, DCM layer, and BOT were 5.88%, 15.95%, and 30.23%, respectively. For Cyanobacteria, the relative contributions to CCF potential in the surface layer and DCM layer in the shelf region were 13.34% and 0.74%, respectively. While in the slope region, the relative contributions to CCF potential in the surface layer, DCM layer, and BOT were 27.15%, 5.87%, and 1.23%, respectively. For Dinophyceae, the relative contributions to CCF potential in the surface layer and DCM layer in the shelf region were 18.37% and 3.30%, respectively. In the slope region, the relative contributions in the surface layer, DCM layer, and BOT were 6.00%, 1.36%, and 0.50%, respectively.

In general, at both study sites, the relative transcript contribution of Bacillariophyta to the CCF of plankton increased with depth, while the relative transcript contribution of Cyanobacteria and Dinophyta decreased with the increase of depth. At the same water depth, the proportion of CCF of Bacillariophyta and Dinophyta was higher at shelf than slope, while the proportion of CCF of Cyanobacteria and O_Stramenopiles was higher at slope than shelf. We also estimated the CCF contribution of major plankton at the order level. In total, no fewer than 117 orders of plankton were detected to actively express the genes involved in CCF, among which the top 10 plankton orders contributed 54.96%–76.84% of all measured transcripts in each sample. The main orders active in CCF were Bacillariales, Pelagomonadales, Prymnesiales, Mamiellales, and Naviculales (Fig. 2B). The proportion of Gymnodiniales, Dinophyta, and cyanobacteria subsection I in CCF decreased with the increase of depth, and the proportion of Naviculales in CCF increased with the increase of depth.

**Fig 3:**
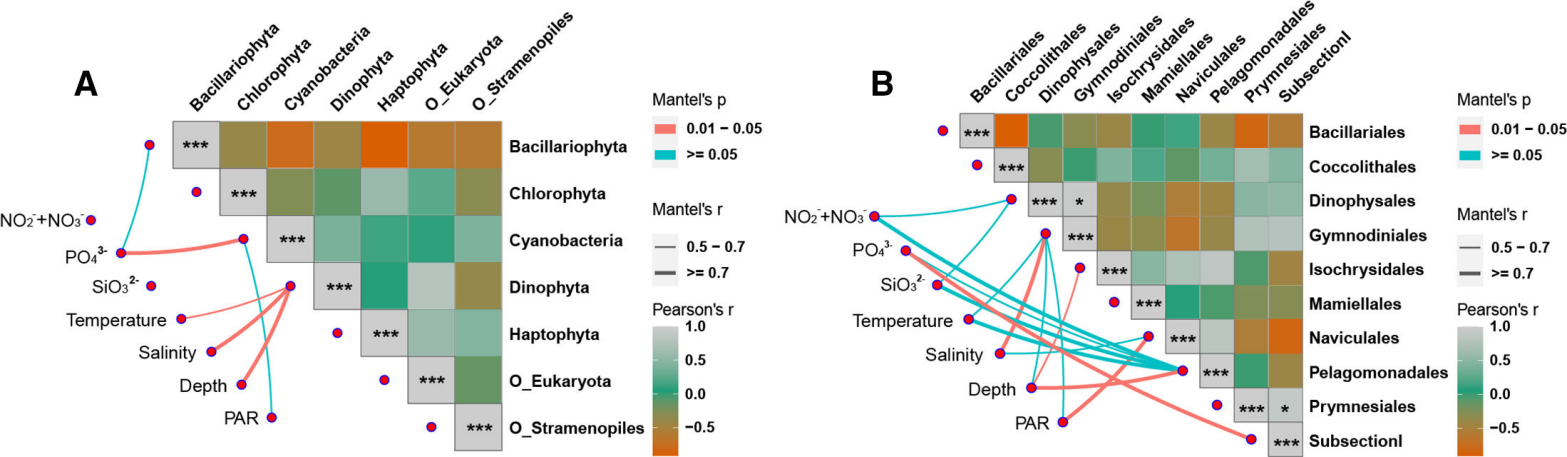
The relationship between CCF potential and environmental factors of major supergroups (**A**) and orders (**B**) of plankton. The thickness of the line represents the correlation between environmental factors and taxon abundance, and the color of the lines represents the significance level of the correlation (see color scale and thickness scale on the upper and middle right). Fill color strength of the squares represents the correlation of selected major taxa, from orange (negative interaction), green, to gray (positive interaction) (see the color scale on the lower right). Asterisks indicate significance (*P < 0.05 and ***P < 0.01).

Furthermore, we examined the relationship of the CCF community structure and environmental factors of different planktons at the supergroup and order levels, respectively. At the supergroup level, the CCF transcript contribution of Bacillariophyta was negatively correlated with the concentration of P in seawater (Fig. 3A). The CCF transcript contribution of Dinophyta was positively correlated with temperature and negatively correlated with salinity and depth. At the order level, the CCF transcript contribution of Gymnodiniales, Isochrysidales, and Naviculales was significantly related to salinity, depth, and photosynthetically active radiation, respectively, while the contribution of Pelagomonadales to CCF transcript pool was positively correlated to N and Si concentrations and temperature and is negatively correlated to depth (Fig. 3B). Based on the gene expression data of light-harvesting proteins and nutrient transporters (Fig. S2), CCF potential in Suessiales, Mamiellales, and Prymnesiales was positively correlated with light energy harvesting and uptake of N and P nutrients. CCF potential of Bacillariales was positively correlated with the acquisition of light energy, P, and Si but not with N nutrient.

### Correlation between CCF and endocytosis gene expressions

**Fig 4:**
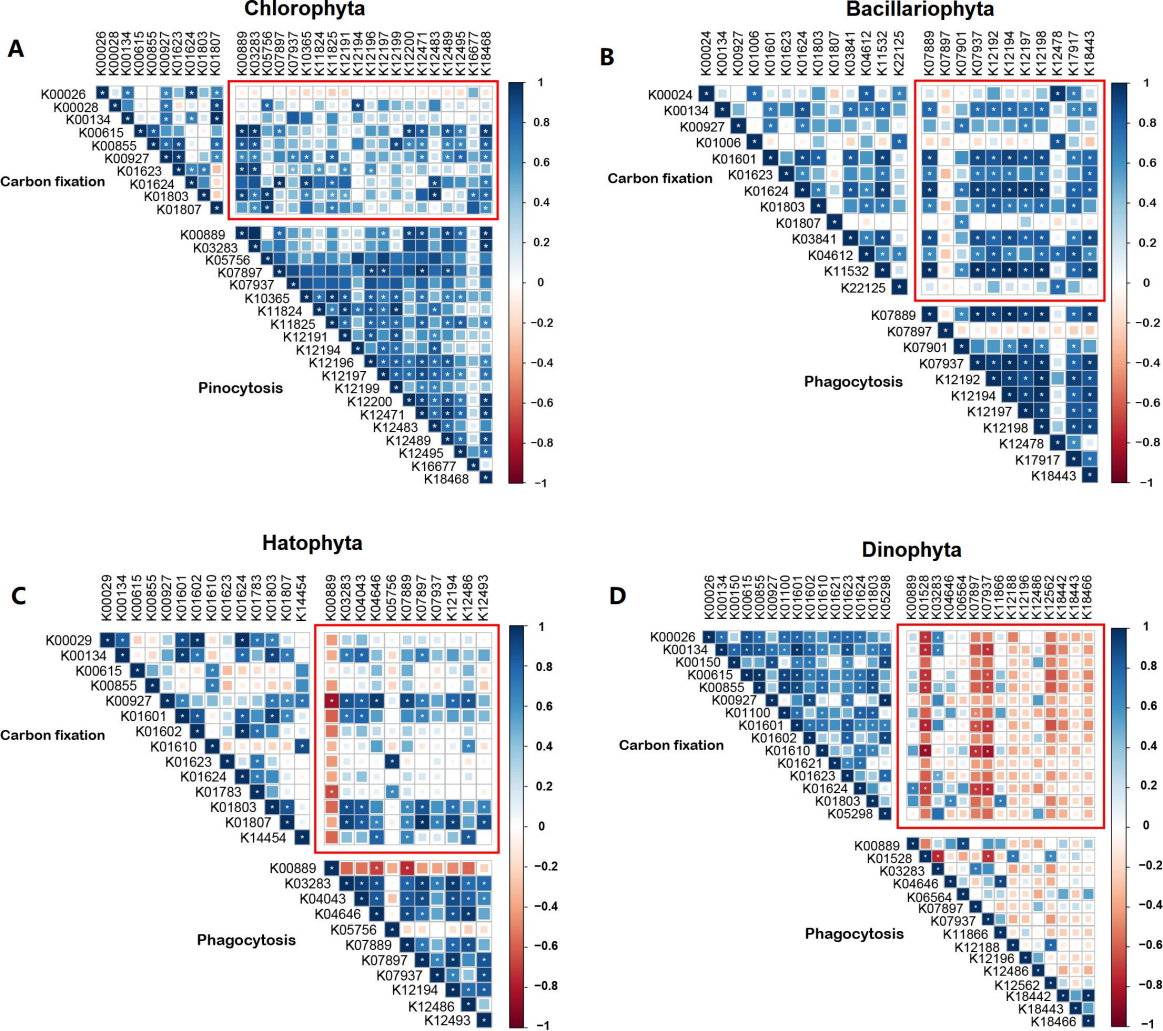
Relationship between the expression of core photosynthesis genes and that of core endocytosis (pinocytosis/phagocytosis) genes in major phytoplankton phyla. The relationships are shown in red frames. Fill color of the squares depicts the sign and degree of correlation according to the scale bar on the right. (A) Chlorophyta; (B) Bacillariophyta; (C) Haptophyta; and (D) Dinophyta. *P < 0.05. KO numbers represent genes, whose names can be found in KEGG (Table S5).

Many protists are mixotrophic, acquiring nutrients through phagocytosis either to support obligate photosynthesis or to promote proliferation with the aid of facultative photosynthesis (22, 23). For these two contrasting trophic groups of protists, a positive or negative correlation between the expression of endocytosis genes and that of carbon fixation genes, respectively, is expected. We analyzed the correlation between the expression of core genes in the carbon fixation pathway and the core genes of endocytosis (indicators of pinocytosis/phagotrophy) in each major CCF-active lineage. We found that the expression of the CCF core genes in Bacillariophyta, Chlorophyta, and Haptophyta was positively correlated with the expression of their endocytosis core genes. The correlation was negative in Dinophyta (Fig. 4). Although the overall two pathways show a specific relationship, it is possible that some of the individual genes in them deviate the overall pattern. For instance, within Chlorophyta, K00026 seems to have a slight negative relationship with all pinocytosis genes, while in Haptophyta, K00889 also has a negative relationship with all carbon fixation and other phagocytosis genes (Fig. 4).

### NCF in different plankton groups and environmental effects

**Fig 5:**
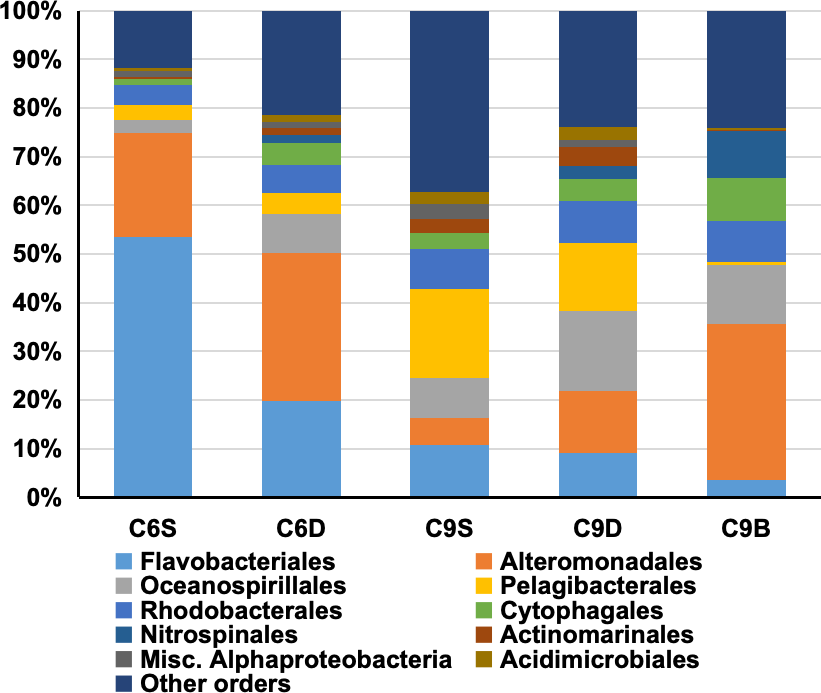
Relative expressional contribution to NCF of different orders of NCF bacteria in five water masses.

NCF was characterized using the expression profiles of the currently recognized five NCF pathways (reductive citric acid cycle, Wood-Ljungdahl pathway, 3-hydroxypropionate bicycle, hydroxypropionate-hydroxybutyrate cycle, and dicarboxylate-hydroxybutyrate cycle). The results of our analyses showed that at both stations NCF was contributed by Proteobacteria, Bacteroidetes, Archaea, and other lineages of bacteria (O_Bacteria). The highest relative expression of NCF genes was in the surface layer of station C6, and the lowest was in the DCM layer at station C9 (Fig. S3A). At the order level, 45 orders of prokaryotes were found to express at least 15 of the NCF pathway genes, among which the main five orders were Flavobacteria, Alteromonales, Oceanospirillales, Pelagibacterales, and Rhodobacterales (Fig. 5). Flavobacteriales contributed more than half of the NCF transcripts on the surface of the C6 station, and its contribution to the NCF transcript pool at the two stations decreased with increasing depth, while the NCF transcript contribution of Alteromonadales, Oceanospirillales, Cytophagales, and Nitrospinales increased with increasing depth (Fig. 5).

**Fig 6:**
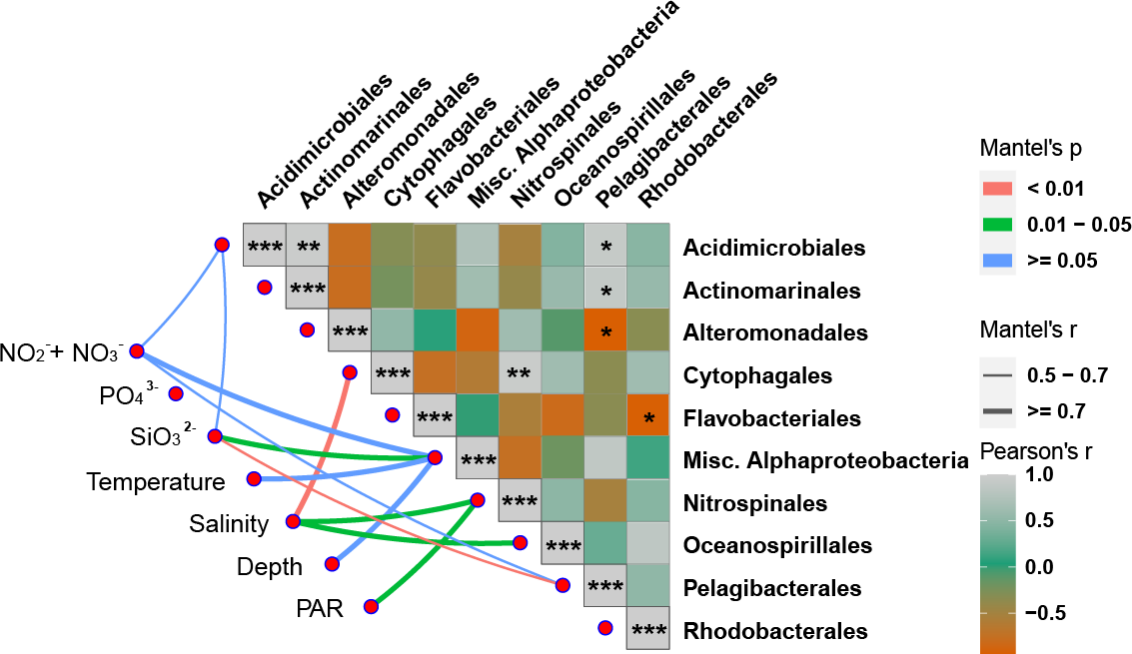
Relationship between relative contribution (%) of NCF transcript pool by 10 major orders of NCF bacteria and environmental factors. The thickness of the line represents the correlation between environmental factors and taxon abundance, and the color of the lines represents the significance level of the correlation (see the color scale and thickness scale on the upper and middle right). Fill color strength of the squares represents the correlation of selected major taxa, from orange (negative interaction), green, to gray (positive interaction) (see the color scale on the lower right). Asterisks indicate significance (*P < 0.05; **0.05 < *P* < 0.01; and ***P < 0.01).

In the correlation analysis between NCF transcript contribution and environmental factors of the 10 major orders, we found that the NCF transcript contribution of Miscellaneous Alphaproteobacteria was positively correlated with temperature, depth, and N and Si concentrations. Nitrospinales’ NCF transcript contribution was positively correlated with salinity and PAR, while Cytophagales and Oceanospirillales’ NCF transcript contributions were affected by salinity. In addition, Pelagibacterales’ contribution to the NCF transcript pool appeared to be negatively affected by Si concentration (Fig. 6).

In addition, by calibrating with size-fraction RNA quantities from the same water sample, we compared the NCF transcript abundances of two size samples in each water layer and found that both large- and small-sized groups of plankton contributed approximately equally (Fig. S3B). However, the NCF transcript abundances of plankton in small-sized samples in the SUR layer of the C6 station and the DCM layer of the C9 station were slightly higher than that of large-sized samples, and the NCF transcript abundances of large-sized samples in other water depths were higher than that of small-sized samples (Fig. S3B).

### The profile of rhodopsin and its relationship with NCF potential

**Fig 7:**
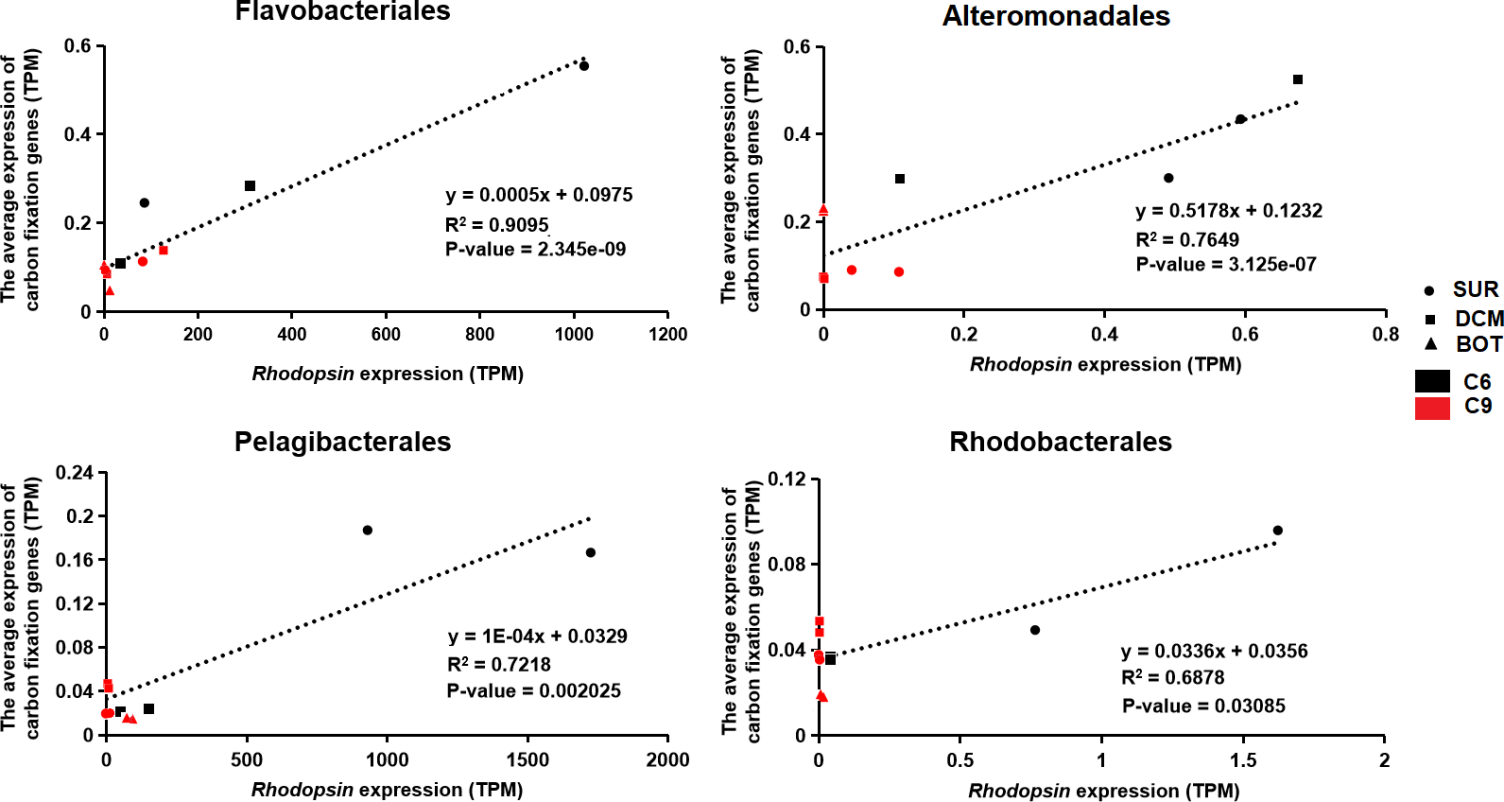
The correlation between the relative expression of NCF genes and the expression of rhodopsin genes in four major orders of NCF bacteria.

**Fig 8:**
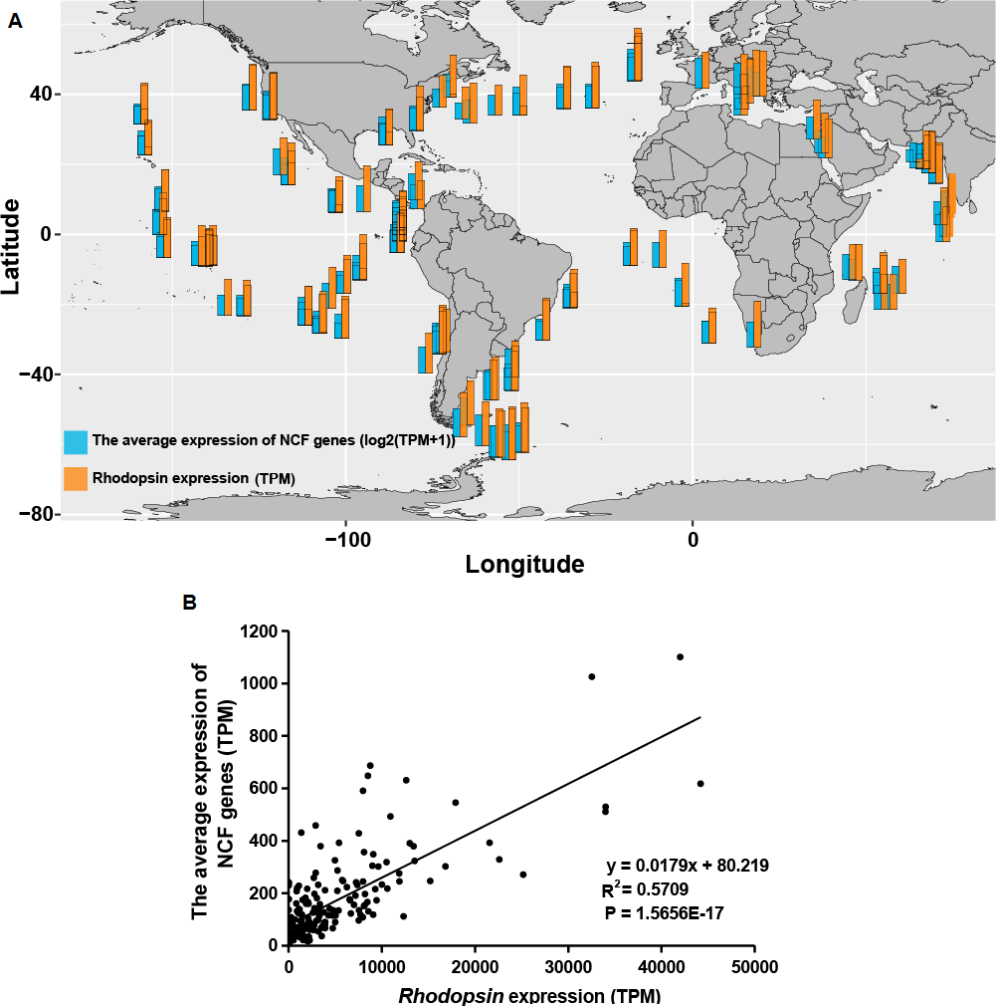
Correlation between the relative gene expression of NCF and the gene expression of rhodopsin in Flavobacteriales in the global ocean. (A) The atlas of NCF gene expression [log2 (TPM + 1)] and rhodopsin gene expression (TPM). (B) Linear correlation between the expression level of Flavobacteriales NCF and rhodopsin.

A high diversity of proton-pump rhodopsin (PPR) was found to be actively expressed. In total, 1,774 rhodopsin unigenes were detected, which were mainly contributed by Proteobacteria, Dinophyta, and O_Bacteria. At the order level, Flavobacteriales and Pelagibacterales were the two most dominant orders that contributed to the expression of rhodopsins (Fig. S4). Among the top six orders of prokaryotes with the highest PPR expression levels, four were carbon fixers involved in NCF: Flavobacteriales, Alteromonadales, Pelagibacterales, and Rhodobacterales. Statistical analysis showed that the expression of PPR was positively correlated with the expression of carbon fixation genes for these four groups of bacteria, with *R*^2^ values ranging from 0.68 to 0.90 (Fig. 7). Furthermore, we used the same method to analyze 187 samples collected from 66 stations in Tara Oceans database (31) and found that the transcript abundance of NCF of Flavobacteria in these samples was significantly correlated with its PPR expression level (Fig. 8).

## DISCUSSION

In this study, we used whole-assemblage metatranscriptome (WAME) sequencing to be able to compare prokaryotes with eukaryotes for common metabolic profiles or physiological functions. Furthermore, using RNA quantity-based calibration, data from separately sequenced size fractions from the same water sample could be integrated to reconstruct the assemblage gene expression profile. This approach enables the estimation of the metabolic contribution of each size group to the overall community in various physiological functions, as represented by the genes expressed in both size groups. For example, it allows for the comparison of free-living bacteria (in the 0.2–3 µm fraction) versus particle-associated (PA) bacteria (in the 3–200 µm fraction) in terms of NCF.

Using this integrative WAME approach, we obtained novel insights into the relative contributions of different lineages to CCF and NCF, environmental effects, and a potential energy source. As is true for any molecular ecological data, the relationship between gene expression and the metabolic activity supported by the encoded proteins may not be linear and could vary significantly between different organisms. Thus, we caution that the lineage-differential metabolic potentials still require experimental verification in the future. There are some potential methods that could be employed: (i) enzymatic assays to measure the activity of specific metabolic pathways or enzymes of interest. This can help validate the metabolic potentials inferred from gene expression data; (ii) stable isotope probing to trace the incorporation of specific isotopically labeled substrates into biomass. This can provide direct evidence of the metabolic activity and substrate utilization of different lineages in the community; and (iii) metabolomics analysis to measure the changes in metabolite profiles and concentrations. By comparing the metabolomes of different lineages, it is possible to validate their metabolic potentials and identify the key metabolites associated with specific functions. Nevertheless, insights obtained from metatranscriptomic studies have been proven highly valuable. For instance, this type of work has led to the discovery of PPR in dinoflagellates (1) and diatoms (7) as a source of energy to cope with nutrient limitation and trypsin in diatoms as a regulator of nitrogen-phosphorus stoichiometric homeostasis (32, 33). The results obtained in the present study shed light on how carbon fixation might vary between different lineages and might be differentially influenced by biotic and abiotic factors, providing bases on which to construct testable hypotheses for further research in the future.

Some hypotheses that can be derived from the results obtained in this study include (i) about biotic factors that influence the relative contribution of different lineages to carbon fixation, for example, it can be hypothesized that specific lineages of free-living (FL) bacteria (in the 0.2–3 µm fraction) may contribute more to NCF than particle-associated bacteria (in the 3–200 µm fraction) do due to the differences in their ecological niches and interactions with other organisms; (ii) abiotic factors that affect the metabolic activity and carbon fixation in different lineages, for instance, it can be hypothesized that environmental conditions such as nutrient availability, temperature, or light intensity may influence the relative contribution of certain lineages to the CCF and NCF pathways; (iii) about the potential that specific lineages may possess unique adaptations for efficient carbon fixation, for example, it can be hypothesized that certain eukaryotic lineages might have evolved specific molecular mechanisms, such as the presence of PPR, to cope with nutrient limitation and enhance their carbon fixation abilities; (iv) about the potential that interactions between different lineages influence carbon fixation dynamics, it can be hypothesized that the presence of specific lineages or the interactions between different organisms in a community may have a positive or negative effect on the carbon fixation rates of the community or the relative contribution of different lineages.

### CCF transcript pool was differentially contributed by different lineages and affected by environmental factors

For CCF, the transcript level of CCF in the continental shelf area was higher than that in the slope area (Fig. 2). This suggests a potentially higher CCF activity in the shelf area, which might be attributed to higher nutrient concentrations there due to greater influences from more nutrient input than in the slope area (26, 34). Indeed, the CCF transcript abundance of the major CCF groups was in most cases positively correlated with nutrient concentrations (Fig. S2).

At the supergroup level, CCF potential was mainly contributed by Bacillariophyta, Cyanophyta, Chlorophyta, Haptophyta, and O_Stramenopiles in our samples overall. Northern SCS summer phytoplankton communities have been found to be dominated by Cyanobacteria, Bacillariophyta, Haptophyta, and Chlorophyta (35, 36). This is similar to the taxonomic profile of CCF plankton in our study. The CCF transcript contribution of plankton was, at least in part, correlated with their abundance, as is often found to be the case (37, 38). In the photic zone, the contribution of Bacillariophyta and Dinophyta to the total CCF of plankton was higher in the shelf area than in the slope area and higher at greater depths. On the contrary, the contribution of Cyanobacteria to the whole plankton’s CCF potential in the shelf area was lower than that in the slope area and decreased with the increase of depth. This trend is consistent with the general understanding that cyanobacteria are predominant primary producers in the major ocean basins (offshore), whereas diatoms and dinoflagellates dominate nearshore waters, due to contrasting nutrient availabilities.

The major CCF contributors at the taxonomic level of the order were generally similar between the shelf and slope areas, including Bacillariales, Pelagomonadales, Prymnesiales, Mamiellales, and Naviculales, but without any cyanobacterial order, suggesting cyanobacterial contributors to CCF were more spread out taxonomically. Despite the shelf versus slope similarity, the CCF transcript abundance of plankton in different orders varied with geographical location and depth. Bacillariales was most transcriptionally active in the shelf DCM layer, Mamiellales was most active in the slope DCM layer, and Dinophysales was most active in the SUR layer. This pattern might reflect niche differentiation among these orders, particularly due to their differential potential to acquire light energy and nutrients. For instance, diatoms favor high inorganic nutrients (39), whereas Mamiellales species prefer to be distributed in the subsurface layer with high chlorophyll *a* concentration (40). Dinoflagellates are more competitive in nutrient-poor environments due to propensity for mixotrophy, and they are versatile in scavenging nutrients (22). In addition, dinoflagellates possess PPR for efficiently harvesting light energy (1), which might help prevent photoinhibition that often occurs on sea surface phytoplankton (41). Indeed, dinoflagellates were actively expressing rhodopsin (Table S3) and phagotrophy genes in the present study.

Taken together, our results indicate that diverse taxa of both cyanobacteria and eukaryotic phytoplankton actively contributed to primary production in our study area, with no taxa being predominant in photosynthetic carbon fixation. Furthermore, the CCF potential of these contributing taxa was differentially influenced by the common environmental conditions such as nutrient and light availability. In addition, dinoflagellate CCF potential might, in part, be supported by phagotrophy as a source of nutrients and PPR as a supplemental source of energy. For instance, in laboratory experiments, *Prorocentrum shikokuense* actively upregulated the phagocytosis pathway under deficiency of inorganic nitrogen nutrients (22) and upregulated rhodopsin under phosphorus limitation (42) and during bloom outbreaks in phosphorus-poor waters (8).

### NCF by both free-living and particle-associated microbes

In the present study, all the five NCF pathways were represented in the whole-assemblage metatranscriptomic data set, indicating that there were five different ways of bacterial NCF in the euphotic zone of the northern South China Sea. Based on the average expression level of complete pathway genes, we found that Flavobacteriales, Alteromonadales, Oceanospirillales, and Pelagibacterales were the dominant NCF contributors in the northern SCS for NCF (Fig. 5). Generally, prokaryotes in small-sized samples are FL, while prokaryotes in large-sized samples are PA, although some FL species might have been retained on the >3 µm fraction due to clogging during filtration. Studies in multiple sea areas have shown that the diversity and abundance of PA bacteria are generally higher than that of FL bacteria (43, 44). In this study, FL and PA prokaryotes contributed nearly equally except in C6_DCM and C9_SUR, where PA prokaryotes’ contribution was higher than FL prokaryotes. Our data suggest that the PA prokaryotes may contribute significantly to non-Calvin microbial carbon fixation in SCS, highlighting their potential importance in this process. Particles might provide a concentrated supply of substrates to support carbon fixation. Our findings in this study demonstrate the potential of non-photosynthetic bacteria to contribute to ocean production, which should be further investigated in other sea areas and verified in laboratory experiments.

### Lineage-differential effects of environmental factors on CCF and NCF

Marine CCF is well known to be affected by environmental factors such as light cycle and light intensity (45, 46), salinity (47), temperature (48), N-, P-, and Si-nutrients (22, 42, 49), and trace metals (50). However, the effects differ between species, and it is challenging to investigate lineage-differential effects in a natural community *in situ* using ecological or physiological methods. In this study, we examined the relationship between the lineage-specific carbon fixation potential (gene expression) and environmental factors at supergroup and order levels and found differential responses of different lineages to environmental factors. The relative expressional contributions of cyanobacteria to CCF were found to be negatively correlated with the phosphate concentration (Fig. 2 and 3), which was probably because cyanobacteria are highly capable of utilizing dissolved organophosphate (51, 52). This is consistent with the previous observation that the abundance of cyanobacteria is positively correlated with the N:P ratio (53). The dinoflagellate proportion of the CCF transcript pool was positively correlated with temperature and significantly negatively correlated with salinity (Fig. 2 and 3). Dinoflagellates are generally known to favor warmer temperatures and have been reported to prefer lower salinity and higher temperature conditions (54). Lower salinities are often linked to freshwater input that brings in organic and inorganic nutrients (55).

Regarding NCF, the relative expressional contribution of Cytophagales, Nitrospinales, and Oceanospirillales was significantly positively correlated with salinity. This suggests that these three orders might be more NCF active in higher salinity environments than other major orders of NCF contributors. In contrast, some orders of bacteria did not show a clear trend in NCF transcript abundance in relationship to environmental factors. Therefore, the NCF potential of different species from the same order can respond differently to environmental factors. As NCF has been understudied, more research is required to unravel how salinity and other environmental factors influence this type of carbon fixation.

In sum, our data in concert indicate that the responses of carbon fixation to environmental factors may be different between phytoplankton and non-photosynthetic bacteria, as well as between taxa within each group of organisms. When species coverage of the genome and transcriptome databases increases in the future, the relationship between carbon fixation activity and environmental factors can be studied at a finer taxonomic level, and more trends of NCF with environmental factors might emerge.

### Role of endocytosis in supporting CCF

Endocytosis is usually divided into three types: phagocytosis, pinocytosis, and receptor-mediated endocytosis due to the different sizes of the material and the mechanism (56). It is increasingly recognized that many phytoplankton are mixotrophic (hence named mixoplankton) (57–59). Diatoms can take in iron ions in seawater through pinocytosis and even capture silicon to support their growth (60–62). Many dinoflagellates can perform phagocytosis to cope with the lack of nutrients in the environment (8, 22). Viruses and commensal microorganisms enter the cells of protists through the endocytic pathway of the protistan hosts (63). Therefore, the endocytosis of these CCF microorganisms might serve as a supplementary nutritional mechanism to support their photosynthesis and carbon fixation or compensate for reduced photosynthetic carbon fixation due to nutrient deficiency. In these two scenarios, a positive and a negative correlation, respectively, is expected for carbon fixation gene expression and endocytosis gene expression. In this study, we analyzed the relationship between photosynthesis and endocytosis of Bacillariophyta, Dinophyta, Haptophyta, and Chlorophyta. The results revealed a positive correlation between the core genes of endocytosis and the core genes of photosynthesis in Bacillariophyta, Haptophyta, and Chlorophyta (Fig. 4). This suggests that these diatoms might be taking in iron and other nutrients through pinocytosis to support their photosynthesis (61). Besides, through phagocytosis, the haptophytes and chlorophytes might be obtaining essential growth factors to promote the photosynthetic growth of the CCF population (64). In sharp contrast, a negative correlation was found in dinoflagellates between the expression of phagocytosis core genes and that of photosynthesis core genes (Fig. 4). This is probably because many dinoflagellates are mixotrophic and use phagotrophy to compensate for the reduction of photosynthesis due to nutrient limitation in the subtropical ocean. Although the overall two pathways show a specific relationship, it is possible that some of the individual genes in them deviate the overall pattern. For instance, within Chlorophyta, K00026 seems to have a slight negative relationship with all pinocytosis genes, while in Haptophyta, K00889 also has a negative relationship with all carbon fixation and other phagocytosis genes. Malate dehydrogenase (K00026) in the carbon fixation pathway of Chlorophyta showed a slightly negative correlation with pinocytosis pathway genes. Malate dehydrogenase is involved in the process of energy metabolism, while pinocytosis requires energy expenditure. The negative correlation of the gene expression of CCF and pinocytosis may reflect the cellular allocation of energy utilization. When cells require more energy, gene expression of pinocytosis may be suppressed, while malate dehydrogenase expression is increased to improve energy supply. This may be the way of energy metabolism regulation in Chlorophyta, which warrants further investigation in the future. In haptophytes, K00889 encodes 1-phosphatidylinositol-4-phosphate 5-kinase (PIP5K), and its negative correlation with carbon fixation and other pinocytosis genes might be because carbon fixation and pinocytosis require energy and resources from the cell. PIP5K may affect the allocation of these limited resources within the cell, leading to negative correlations between its expression and the genes involved in carbon fixation or pinocytosis.

To obtain support from a wider seascape, we analyzed the correlations for chlorophytes and dinoflagellates using the metatranscriptomic data from the Tara Oceans expedition (https://tara-oceans.mio.osupytheas.fr/ocean-gene-atlas/). We found a similar positive correlation for chlorophytes between the expression of core genes in CCF and endocytosis (Fig. S5A). For dinoflagellates, however, the result indicated a positive correlation instead of a negative one (Fig. S5B). The disparity between our data and the Tara Oceans data for dinoflagellates is probably not so surprising, again because the global ocean sampling in the latter might have covered more photosynthetic species than our subtropical smaller geographic range. Alternatively, the wider range of environmental conditions in the Tara Oceans expedition may mask the relationships within a smaller range of environmental conditions in the northern South China Sea.

As the relationship between endocytosis and photosynthetic carbon fixation is still underexplored, further research is needed. In particular, direct evidence needs to come from laboratory experiments on isolates of these species or fieldwork using a combination of microscopic, isotope tracing, and omics analyses.

### Rhodopsin as a potential energy source for NCF in bacteria

As discussed earlier, studies in recent years have revealed that many prokaryotes in the ocean are engaged in carbon fixation activities through five NCF pathways (65). However, the source of energy to support NCF remains elusive. While there is chemical energy from organic carbon metabolism or the energy from the oxidation of inorganic compounds (66), another potential source is light energy harnessed by PPR. This structurally very simple and hence highly efficient machinery includes all-trans retinal coupled with an opsin protein and absorbs light energy from the blue-green waveband to pump protons outside of the cell membrane, thus creating a proton gradient to drive the synthesis of ATP (67, 68). PPR is therefore a photosystem-independent solar energy converting mechanism. PPR is widespread in marine bacteria, in which the energy harnessed facilitates the survival and growth of the bacteria in nutrient-limited environments (69, 70). Therefore, rhodopsin expression was analyzed for the four NCF bacterial orders with the highest relative PPR expression: Flavobacteriales, Alteromonadales, Pelagibacterales, and Rhodobacterales. The results showed that the four orders with the highest NCF potential were also the four orders with the highest rhodopsin gene expression, and the rhodopsin expression level of these four orders exhibited significantly positive correlations with their NCF gene expression (Fig. 7). To explore whether the same correlations occur in other parts of the global ocean, we mined the metatranscriptome database of the Tara Oceans expedition. Indeed, the same result was found in *Flavobacterium* (Fig. 8). Based on all these results, it is likely that rhodopsin is a potential energy source for NCF in some lineages of NCF-active bacteria.

### Conclusion

There has been an increase in large-scale studies using meta-omics to understand the metabolic performance of microorganisms in the ocean (13), but relatively few have dealt with lineage-specific carbon fixation, compared CCF with NCF, and examined their respective relationships with environmental effects. This is particularly true for the South China Sea, a large subtropical continental marginal sea. Our first attempt to catalog functional genes in the China Seas documented over 4.4 million unigenes. This will be useful baseline data for future research on plankton metabolic pathways *in situ* in this area and a potentially valuable resource for research in other parts of the global ocean. Previous metatranscriptomic studies have tended to focus on either eukaryotes or prokaryotes, and our whole-assemblage metatranscriptomics approach includes both groups. This allows us to make a comparative analysis of the contributions of prokaryotes and eukaryotes to common physiological processes at the level of gene expression and better understand the interaction between eukaryotes and prokaryotes. At the transcriptomic level, we have assessed the contribution of the major carbon-fixing plankters in different water layers in the shelf and slope areas of the northern South China Sea. The results indicate that Bacillariales, Pelagomonadales, Prymnesiales, Mamiellales, and Naviculales were the major contributors of the CCF transcript pool, while Flavobacteriales, Alteromonadales, Oceanospirillales, and Rhodobacterales were most transcriptionally active in NCF. CCF and NCF potential capacities were higher at the shelf and differentially affected by environmental factors depending on lineages. Furthermore, our data suggest that endocytosis may promote CCF in diatoms, chlorophytes, and haptophytes, while it may complement CCF in dinoflagellates. NCF in Flavobacteriales, Alteromonadales, Pelagibacterales, and Rhodobacterales appears to be energetically supported by PPR. Testable hypotheses can be developed based on our findings for verification or further inquiries of related questions. Moreover, this study sheds light on the physiological characteristics of different taxa of planktonic microorganisms in a subtropical marginal sea and provides a reference for future studies on the response of plankton to environmental factors in different geographical locations.

## MATERIALS AND METHODS

### Field sampling

During a research cruise in the period of 6–12 August 2016, 20 samples were collected at C6 (117.46 °E, 22.13 °N) and C9 (117.99 °E, 22.69 °N) stations from the northern South China Sea, separated into the picoplankton/nanoplankton (0.2–3 μm) and microplankton (3–200 μm) organismal size fractions. Stations C6 (77 m deep) and C9 (1,369 m deep) were located on the shelf and slope of the northern South China Sea, respectively. Sampling targeted three depth layers, which encompassed distinct physicochemical conditions: surface, deep chlorophyll maximum, and bottom of photic zone layers. For each sample, 40–50 L seawater from more than three Niskin bottles (there are 12 Niskin bottles on a CTD rosette, and the volume of each Niskin bottle is 12 L) was first filtered through 200 µm blotting cloth to remove large planktons. Then, the seawater was serially filtered through two size fractions (0.2–3 and 3–200 μm) using 142 mm polycarbonate membranes (Millipore, Billerica, MA, USA), each sample taking about 20 min. Finally, the sample-carrying filters were divided into four quarters: one being stored in a 2 mL tube containing lysis buffer (0.1 M EDTA and 1% SDS) for DNA work and the other in Trizol reagent (Molecular Research Center, Inc., USA) for RNA work. All samples were immediately frozen in liquid nitrogen onboard until processing upon return to the laboratory. Two replicate samples were collected from each condition; a total of 20 samples were collected (Fig. 1).

### Measurements of physical and chemical parameters

Temperature, salinity, and depth were measured using a CTD profiler (SBE 17plus V2, Sea-Bird Scientific, USA). A series of nutrients (NO_3_^−^+ NO_2_^−^, PO_4_^3−^, and SiO_3_^2-^) were measured using a continuous flow analyzer, and all measurements were performed on triplicate samples. The light intensity of each water layer was calculated based on remote sensing data and a reported computational algorithm (71).

### Sample processing and metatranscriptomic sequencing

RNA was extracted as previously described (32). Shortly, each of the samples was mixed with a 1:1 mixture of 0.5 and 0.1 mm-diameter zirconia/silica beads (Biospec, USA) and beat at a rate of 6 m/s on a FastPrep-24 bead mill (MP Biomedicals, USA) for three times to ensure complete cell breakage. Each bead beating step lasted for 60 s, and then the centrifuge tubes were placed on ice for a 3-min cold bath, before proceeding to the next bead beating step. RNA was extracted following the TRI Reagent protocol coupled with the Direct-zol RNA columns, essentially as reported previously (1). DNase 1 was used to remove DNA from the total RNA. RNA concentration was measured using a NanoDrop ND-2000 Spectrophotometer, and integrity was assessed using RNA 6000 Nano LabChip Kit in microcapillary electrophoresis (Agilent 2100 Bioanalyzer, Agilent Technologies, Australia). Samples with an RNA integrity number (RIN) ≥ 6.0 were used for metatranscriptome sequencing, and all of our samples in this study satisfied RIN ≥ 6.0. One microgram RNA from each sample was subject to ribosomal RNA removal using a Ribo-Zero rRNA Removal Kit (Plant Leaf), a Ribo-Zero rRNA Removal Kit (Plant Seed/Root) (Illumina, San Diego, CA, USA), and a TIANSeq rRNA Depletion Kit (Bacteria) (TIANGEN Biotech, China) for whole-assemblage (prokaryotes + eukaryotes) metatranscriptome sequencing. mRNA (i.e., rRNA-depleted RNA) was then fragmented with First Strand Synthesis Reaction Buffer and Random Primer Mix (2×) at 94°C for 10 min, and first-strand cDNA was synthesized using ProtoScript II Reverse Transcriptase, and the second-strand cDNA was synthesized using Second Strand Synthesis Enzyme Mix. The double-stranded cDNA was purified, end repaired, and ligated to adaptors. Fragments of about 400 bp (with an approximate insert size of 250 bp) were selected and sequenced on the Illumina HiSeq 4000 instrument (Illumina, San Diego, CA, USA). Each sample had two or three technical replicates and totally produced 881 Gb of raw data; each sequencing yielded 55,123,252–147,337,262 reads (Table S4), with a total of 5,667,140,490 reads.

### Transcriptome analysis and gene expression quantification

Raw reads were processed by removing adaptors, reads with >5% ambiguous bases (N), and low-quality reads (>20% bases with quality value < 20) using SOAPnuke software (v1.5.6) (72) with parameter setting -| 20 -q 0.2-n 0.05 -Q 2. Reads from each sample contained <10% ribosomal RNA, which were discarded. For the remaining clean reads, we used Trinity (v2.0.6) (73) to perform *de novo* assembly for each sample, then used Tgicl (v2.0.6) (74) to remove redundancy for all samples of C6 and C9, respectively. The parameters of Trinity were as follows: (i) group-pairs_distance 500, (ii) min_contig length 200, (iii) min_kmer_cov 2, (iv) min-glue 2, (v) bfly_opts -V 5, (vi) edge-thr = 0.1, (vii) stderr, and (viii) SS_lib_type RF. The parameters of Tgicl were as follows: (i) a minimum of 95% identity between the contigs, (ii) a minimum of 35 overlapping bases, (iii) a minimum score of 35, and (iv) a maximum of 20 unmatched overhanging bases at the sequence ends. The unigene sets from all samples were clustered by using cd-hit (v4.8.1) (75) (-c 0.95 -aS 0.9) to generate the final unigene set (Unigene) for downstream analysis, and the sequence similarity was set at 95%. We used BLASTN (76) for NT annotation, blastx (76) or Di for NR, KOG, KEGG, and Swissprot annotation, blast2go (77) and NR annotation results for GO annotation, and InterProScan 5 (78) for InterPro annotation. The thresholds of BLASTN, blastx, blast2go, and interprocan5 were all e value < 10^−5^, and the top1 was selected as the annotation result. Bowtie2 (79) was used to match clean reads to Unigene, and then Salmon v0.9.1 (80) was used to calculate the gene expression levels of each sample. In the subsequent analysis, we excluded unigenes whose TPM (transcripts per kilobase of exon model per million mapped reads) was less than 0.1 across all 20 samples.

### Identification of key genes in different samples

Key genes in carbon fixation were identified from the WAME data set based on functional annotations. The expression contributions of these genes were visualized using Circos (http://mkweb.bcgsc.ca/tableviewer/visualize/).

### Standardization of gene expression in specific taxonomic groups

While comparing gene expression differences in the same taxonomic group between sample groups, gene expression at the taxonomic group level re-normalized as transcripts of the specific gene from the specific taxonomic group per million of total mapped reads of the taxonomic group was used.

### Statistical analysis

We analyzed correlations for genes with non-zero expression in at least 6 out of our 20 samples. Correlation between the expression of CCF core genes and endocytosis core genes was visualized in R using packages ggplot (81). Pearson correlation was analyzed between the expression of NCF and that of PPR in bacterial lineages in which PPR expression was high. Mantel tests were conducted to determine the correlations between the gene expression and environmental factors using the “ggcor” package.

### Quantification of the relative activity of carbon fixation

Based on the annotations of the KO database, we used the TPM value of the *RuBisCO* gene to represent the CCF activity of the lineage (supergroup) (82). Besides, we counted the sum of the TPM values of all unigenes on the NCF pathway for individual lineages of interest and calculated the average TPM value of each gene in the pathway. The average value was used to represent the NCF activity of the lineage (supergroup). The greater the TPM value, the greater the activity of carbon fixation.

### Calibration for size fraction data set to enable comparison

Large (3–200 µm) and small (0.2–3 µm) size fractions were sequenced separately, making it impossible to directly compare the contributions of these two size groups to the community in various metabolic processes at the transcriptional level. To overcome the problem, we multiplied TPM of a functional gene in a size fraction with the total RNA extracted from the size fraction sample, then divided the product by the total RNA extracted from both size fractions from the same volume of water sample, i.e., TPM_small_ × RNA_small_ per L/(RNA_small_ per L + RNA_large_ per L) and TPM_large_ × RNA_large_ per L/(RNA_small_ per L + RNA_large_ per L). These allow the estimation of the contribution of a small-sized plankton or a large-sized plankton to the whole assemblage in the function represented by the gene (e.g., *RuBisCO*).

### Search for rhodopsin expressed in the global ocean

To understand the expression of rhodopsin in the global ocean, we examined their occurrence in the Tara Oceans metatranscriptome data set. The rhodopsin domain (Pfam ID: PF01036) identified from the rhodopsin protein was used as a query to search in the data sets. For the Tara Oceans data set from all major oceans except the Arctic during 2009–2013, hmmsearch was carried out using the MATOU-v1 catalog with a threshold of 1e^−5^ to study their expression and biogeography.

## Data Availability

The data that support the findings of this study have been deposited in CNGB Sequence Archive (CNSA) of China National GeneBank DataBase (CNGBdb) (https://db.cngb.org/cnsa/) under the accession number CNP0001483. The spreadsheet of the final unigene set alongside their taxonomic and functional annotations, and gene expression levels of each sample in terms of raw read counts, has been uploaded to our laboratory website (http://sampgr.org.cn/index.php/download) and is available for public download.

## SUPPLEMENTAL MATERIAL

Supplemental figures spectrum.02177-23-s0001.docx Fig. S1 to S5.

Supplemental tables spectrum.02177-23-s0002.xlsx Tables S1 to S5.

ASM does not own the copyrights to Supplemental Material that may be linked to, or accessed through, an article. The authors have granted ASM a non-exclusive, world-wide license to publish the Supplemental Material files. Please contact the corresponding author directly for reuse.
