# Supplementary material for: In situ community transcriptomics illuminates CO2-fixation potentials and supporting roles of phagotrophy and proton pump in plankton in a subtropical marginal sea
Source: Microbiol Spectr. 2024 Feb 6;12(3):e02177-23. doi: 10.1128/spectrum.02177-23 (PMC10913738; doi:10.1128/spectrum.02177-23)
Supplement: Supplemental figures — Fig. S1 to S5. [file spectrum.02177-23-s0001.docx]

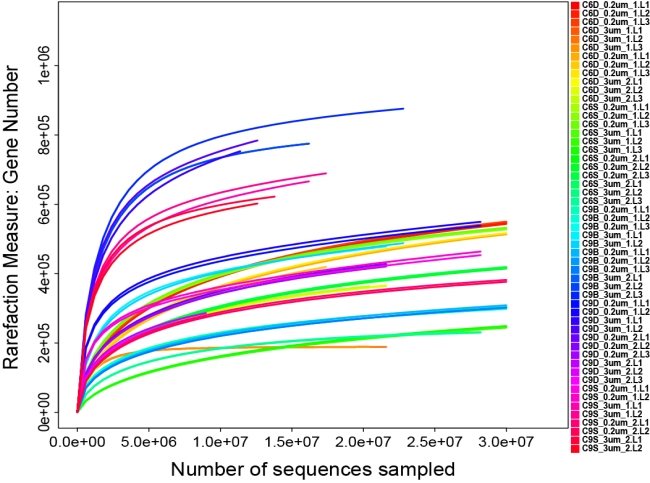


**Fig. S1** Rarefaction curves of detected genes from metatranscriptome sequencing output showing that sequencing scale was nearly saturated. Each plankton sample has 2 to 3 sequencing replicates.


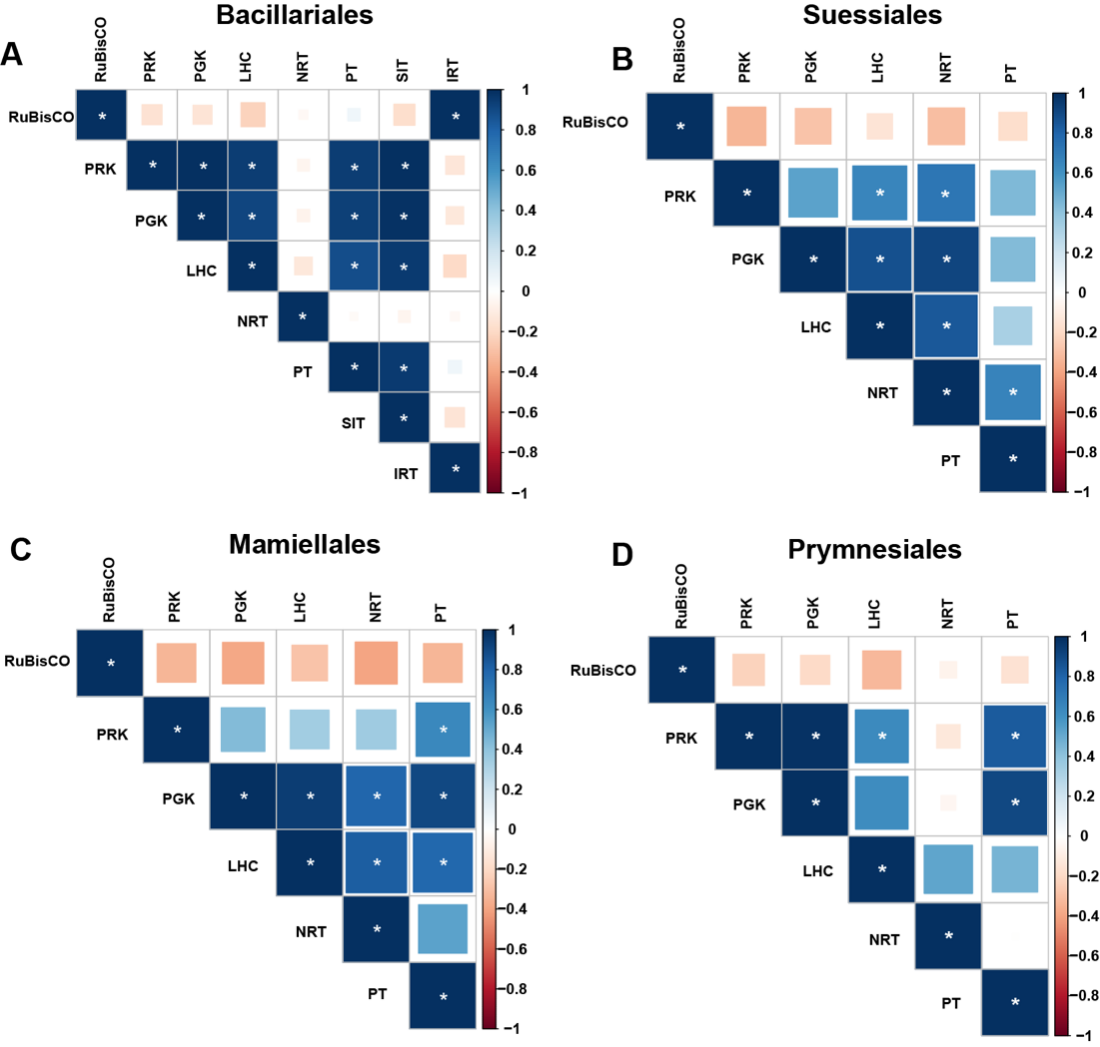


**Fig. S2** The relationship between Calvin carbon fixation (CCF) activity and nutrient transporters. PRK: phosphoribulokinase, PGK: phosphoglycerate kinase, LHC: light-harvesting complex-like protein, NRT: nitrate/nitrite transporter, PT: phosphate transporter, SIT: Silicon transporter, IRT: Iron transporter. * means P < 0.05. Fill color strength of the squares represents the correlation of selected major taxa, from orange (negative interaction), white, to blue (positive interaction) (see color scale on the right).


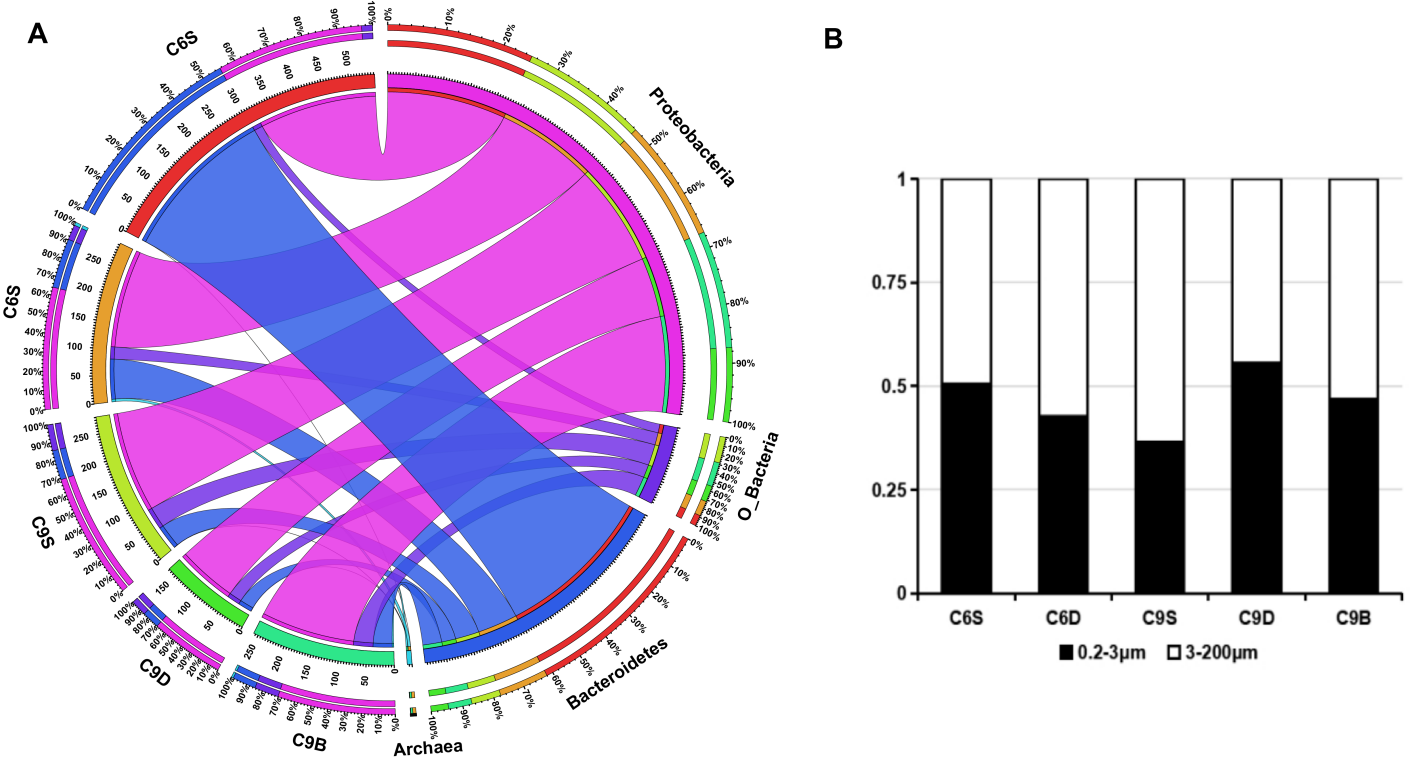


**Fig. S3 The relative activity of non-Calvin carbon fixation (NCF) in five water layers.** A, The relative activity of non-Calvin carbon fixation (NCF) in different water layers and the proportion of contribution by major NCF lineages. The inner circle of numbers are the average expression of NCF pathway genes in the specified station and depth, as proxy of relative activity of NCF. B, Relative contribution (%) of non-Calvin carbon fixation (NCF) by two size fractions.

**
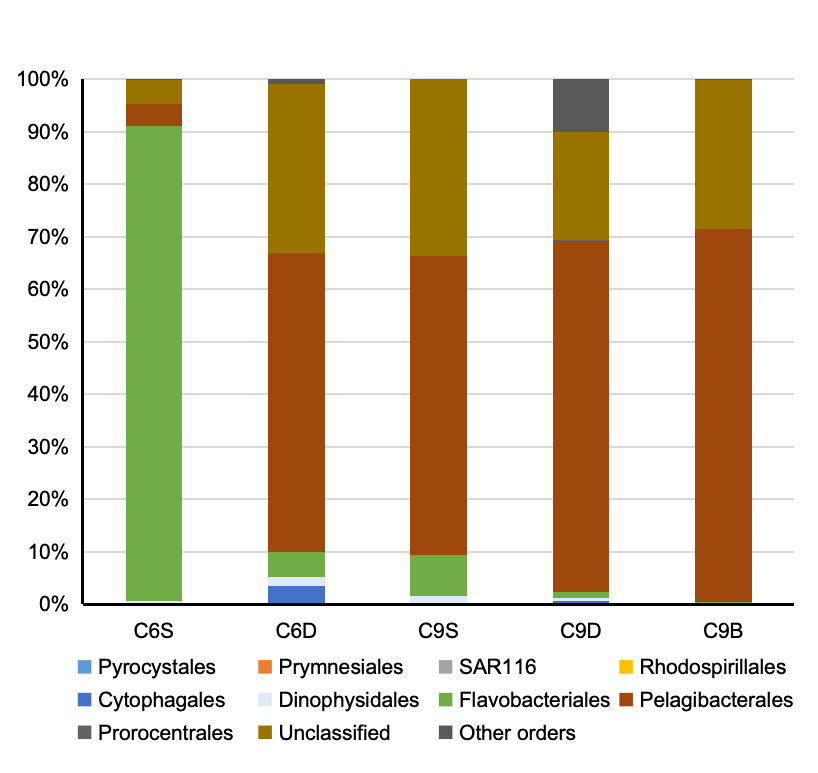
**

**Fig. S4** Expression of rhodopsin from different orders**.**


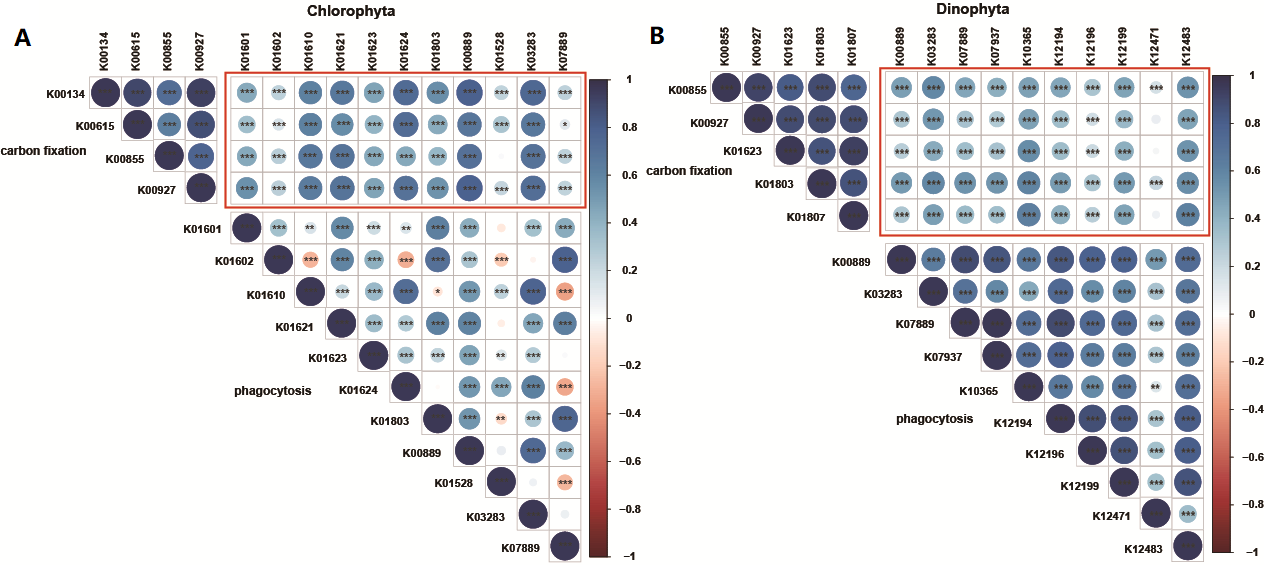


**Fig. S5** Relationship between expression of core carbon fixation genes and that of core endocytosis (pinocytosis/phagocytosis) genes in Chlorophyta and Dionphyta in the global ocean. A, Chlorophyta. B, Dionphyta. The relationships are shown in red frames. Fill color of circles depicts sign and degree of correlation according to the scale bar on the right. *P < 0.05, **P < 0.01, ***P < 0.001. KO numbers represent genes, whose names can be found in KEGG.
